# Effects of copaiba oil on dermonecrosis induced by *Loxosceles intermedia* venom

**DOI:** 10.1590/1678-9199-JVATITD-1493-18

**Published:** 2019-04-25

**Authors:** Mara Fernandes Ribeiro, Felipe Leite de Oliveira, Aline Moreira Souza, Thelma de Barros Machado, Priscilla Farinhas Cardoso, Andrea Patti, Angélica Silveira Nascimento, Cláudio Maurício Vieira de Souza, Sabrina Calil Elias

**Affiliations:** 1 Laboratory of Pharmacology; Department of Pharmacy and Pharmaceutical Administration; School of Pharmacy; Fluminense Federal University; Niterói - RJ, Brazil.; 2 Laboratory for Cellular Proliferation and Differentiation; Institute of Biomedical Sciences; Federal University of Rio de Janeiro; Rio de Janeiro, RJ, Brazil.; 3 Laboratory for Veterinary Clinical Pathology; Department of Pathology and Veterinary Clinics; School of Veterinary Medicine; Fluminense Federal University; Niterói - RJ, Brazil.; 4 Laboratory of Physiochemical Quality Control; Department of Pharmaceutical Technology; School of Pharmacy; Fluminense Federal University; Niterói - RJ, Brazil.; 5 Laboratory of Arthropods; Scientific Directorship; Vital Brazil Institute; Niterói - RJ, Brazil.; 6 Biotherium; Scientific Directorship; Vital Brazil Institute; Niterói - RJ, Brazil.

**Keywords:** Loxosceles intermedia, venom, skin lesion, copaiba oil, topical treatment

## Abstract

**Background:**

Accidents caused by spiders of the genus *Loxosceles* constitute an important public health problem in Brazil. The venom of *Loxosceles sp* induces dermonecrosis at the bite site and systemic disease in severe cases. Traditional medicine based on plant-derived products has been proven to reduce the local effects of envenomation. The present study verified the healing effects of copaiba oil on lesions induced by the venom of *L. intermedia.*

**Methods:**

Cutaneous lesions were induced on the backs of rabbits by intradermal injection of *L. intermedia* venom. Copaiba oil was applied topically 6 hours after injection; the treatment was repeated for 30 days, after which animal skins were removed and processed for histopathological analysis. Blood samples were also collected before and 24 hours after venom inoculation to measure the hematological parameters.

**Results:**

Compared to the control group, the platelet count was reduced significantly in all groups inoculated with venom, accompanied by a decreased number of heterophils in the blood. The minimum necrotic dose (MND) was defined as 2.4 μg/kg. Topical treatment with copaiba oil demonstrated a differentiated healing profile: large skin lesions were observed 10 days after venom inoculation, whereas formation of a thick crust, without scarring was observed 30 days after venom inoculation. Histopathological analysis showed no significant difference after treatment. Nevertheless, the copaiba oil treatment induced a collagen distribution similar to control skin, in marked contrast to the group that received only the spider venom injection.

**Conclusions:**

We conclude that copaiba oil may interfere in the healing process and thus propose it as a possible topical treatment for cutaneous lesions induced by *L. intermedia* venom.

## Background

The venom of spiders belonging to the genus *Loxosceles* produces a characteristic set of symptoms known as loxoscelism. Currently, loxoscelism has been hypothesized to be a multifactorial process involving a direct action of venom on the inflammatory response [1-6]. The main characteristic of envenomation is dermonecrosis at the bite site that is manifested initially by direct and degenerative effects of the venom components on the cell membrane, basement membrane, and extracellular matrix resulting in drastic tissue damage [7].

The early phase of loxoscelism is highlighted by local edema and erythema within 6 hours of the spider bite. Subsequently, the lesion evolves into an ecchymotic area about 24 to 36 hours after the accident. After 5 to 7 days, the necrotic lesion reaches maximum area and develops a dry crust; it is occasionally associated with secondary infection. The necrotic scab falls off 2 to 3 weeks after the accident, leaving an ulcer. The terminal evolution of loxoscelism can induce severe intravascular hemolysis associated with acute anemia, jaundice, and hemoglobinuria of varying degrees. Fatalities are rare, but frequently correlated with acute renal failure [8, 9].

Treatment of loxoscelism is still a controversial subject. The effectiveness of the antivenom serum in neutralizing local effects varies in different therapeutic approaches, especially for the skin lesion [10]. Therefore, there is no consensus as to its efficacy in reversing the local effects, while the ideal interval between the bite and its management that results in good recuperation from the injury. However, antivenom serum is indicated at any time in the case of hemolysis [11, 12].

The low efficacy of the treatment has been attributed to poor understanding of action mechanisms of *Loxosceles* venom [13]. Several treatment protocols that have been proposed and tested for bites by *L. intermedia* include dapsone, corticosteroids, antibiotics, and antivenom. Dapsone limits neutrophil migration and infiltration at the site of the bite [14]; corticosteroids produce a potent anti-inflammatory effect [15]; and antibiotics prevent secondary infections [16]. However, this polytherapy is not completely effective in reducing skin lesions and restoring the affected tissue. In many situations, dermonecrosis is so extensive that it requires skin grafts [9].

The strategies already employed to treat skin lesions include phytotherapy and popular medicinal plant products. Indeed, the effectiveness of some herbal agents used in traditional medicine has been evaluated and confirmed by researchers worldwide [17, 18]. For instance, specialists in wound healing have shown great interest in the use of copaiba oil, a popular medicine in Brazil, extracted from trees of the genus *Copaifera* (Leguminosae-Caesalpinioideae family) to treat scarring [19, 20, 21]. Its property of healing wounds and ulcers is attributed to the presence of medicinally important active components [22]. Copaiba oil is popularly used, especially in the Amazon, as an anti-inflammatory, healing, and antiseptic product that can be administered orally, topically, or vaginally [19]. This oil stands out for its therapeutic properties not only in the Amazon region, but also in northeastern Brazil. Moreover, it is also exported owing to its broad indications for such diseases as cystitis, bronchitis, chronic diarrhea, rheumatism, and psoriasis [23-26].

Sesquiterpene hydrocarbons are the major compounds derived from *Copaifera* oleoresins, the most dominant one being β-caryophyllene, which accounts for more than 90% of the total composition [27]. Sesquiterpenes are responsible for many of the pharmacological activities of copaiba oleoresins. However, some studies correlate increased anti-inflammatory activity with the presence of high levels of diterpenes [28, 29].

No study exists on the potential use of plants with healing effects as therapeutic tools for local manifestations induced by the venom of *Loxosceles sp*. In this context, any scientific evidence describing the healing effects of plant derivatives on skin lesions by loxoscelism would pave the way for the development of topical medicines with wide applicability and benefits for those injured by *Loxosceles* spiders. Thus, the present study evaluated the healing effects of copaiba oil on dermonecrosis induced by the venom of *L. intermedia*, also known as the brown spider.

## Methods

### *L. intermedia* Venom 

The venom of *L. intermedia* was obtained from the Laboratory of Arthropods of Instituto Vital Brazil from adult specimens of *L. intermedia*. The spiders were made to fast for a week followed by extraction of the crude venom.

### Animals

Adult male albino rabbits (*Oryctolagus cuniculus*), weighing 2.5 ± 3.0 kg, were used for *in vivo* tests. These were obtained from the animal colony at the Vital Brazil Institute. All rabbits were fed food and water *ad libitum* under a 12-hour light-dark cycle at 22 ± 2°C throughout the experiment. Venom of *L. intermedia* was injected intradermally into the back of rabbits to induce cutaneous lesions.

### Plant Material

The *Copaifera spp.* oil (copaiba oil) was acquired from a pool of individuals from eastern Amazon (2º 08' 14''-2º 12' 26'' S and 48º 47' 34''-48º 14' W, at an elevation of 16 m above sea level), state of Pará, Brazil. The oil was extracted using the method followed by the local population: Drilling of the trunk of the *Copaifera* tree and insertion of a PVC cannula, through which the oil flows. After the extraction is finished, the hole is sealed with the use of clay. A total of 1 mL of copaiba oil was administered topically at the site of injury.

### Gas Chromatography-Mass Spectrometry Analysis of Sesquiterpenes in *Copaifera spp.* Oleoresin

The sesquiterpenes present in the *Copaifera spp.* oleoresin were analyzed in a gas chromatograph coupled to a mass spectrometer (GC-MS, QP2010SE; Shimadizu). The following chromatographic conditions were used: Rtx-5 capillary column (30 m × 0.25 mm × 0.25 µM MMID); helium as carrier gas at a flow rate of 1.5 mL/minute, oven temperature maintained at 120°C for 2 minutes followed by an increase of 3°C/minute to 160°C for 2 minutes. It was then increased by 8°C/minute to a final temperature of 290°C for 5 minutes; injector temperature of 270°C; and detector temperature of 290°C, operating at 40 to 400 m/z scan mode with electron impact of 70 eV. A volume of 1.0 µL with 1:20 split ratio was injected into the column. The results obtained from analysis of retention times of peaks in the sesquiterpene chromatograms were compared to the data reported in the literature. We also compared the fragmentation spectra of sesquiterpenes with similarity over 90% to those contained in the NIST Library 05.

### Minimum Necrotic Dose Determination

To determine the minimum necrotic dose (MND), rabbits were divided into three groups, each consisting of four rabbits. The backs of these rabbits were first shaved, following which they were inoculated with increasing doses of venom (1.2, 2.4, and 12.0 μg/kg. Cutaneous lesions were observed at 6, 24, and 72 hours after injection. The MND was defined as the lowest dose of venom capable of inducing a necrotic area of at least 1 cm^2^ in 72 hours in 100 % of animals.

### Experimental Groups

The animals were separated into three groups (*n* = 4 per group) as follows: control, venom, and venom plus topical copaiba oil treatment. Each group underwent the same routine observation adopted for calculating the MND. The control group received an intradermal injection of 200 µL of physiologic saline solution (PSS). All other groups received intradermal injection of *L. intermedia* venom at twice the MND determined in a final volume of 200 µL. Topical application of copaiba oil was carried out 6 hours after venom injection and repeated daily for 30 days. Each application using 1 mL of the product completely covered the wound area. During other days of treatment, the remaining product was removed with saline jets, without compromising the newly formed tissue. After washing with 0.9% saline, the product was applied as described above. On days 3, 10, and 30 after the spider venom inoculation and respective treatments, animals were euthanized in a CO_2_ chamber, and skin samples were obtained from the lesioned sites for histopathological analysis according to the procedures described below.

### Blood Analysis

At the beginning of the experiments, blood was collected from rabbits’ ear vein (time 0), prior to inoculation of venom or PSS. After 24 hours, a fresh blood sample was collected from all animals to measure hematological parameters, such as total leukocytes and platelets, using an automatic counter (CC530-CELM). Differential counting was performed on hematological glass slides.

### Macroscopic Lesion Analysis

Macroscopic lesions were analyzed by measuring the wound area. For this, photomicrographs of the lesions were obtained at 0 and 6 hours, and 1, 3, 10, 15, and 30 days after venom inoculation to monitor the healing process. These images were generated using a digital camera (Sony Cyber-Shot DSC-W350, 14.1 mega pixels), which was kept at a constant distance from the tripod base. The injury area was evaluated using the software Image-Pro (unpaid).

### Histopathological Analysis and Skin Processing

Animal skins in the control group and the venom group, containing the venom-induced lesion area, were removed and processed for histopathological analysis. All samples were fixed in 10% paraformaldehyde in a phosphate buffer (pH 7.4) for 48 hours and subsequently dehydrated in increasing concentrations of ethanol. These were next embedded in paraffin, sectioned and placed on slides (5 microns) where they were stained with hematoxylin-eosin and picrosirius red.

### Total Collagen Determination

To quantify the total collagen present in the samples, hydroxyproline was measured using the adapted methodology recommended by the Association of Analytical Communities (AOAC). The skin was macerated and then hydrolyzed with 1 mL of 6 M hydrochloric acid per 0.01 g of skin (maximum 0.08 g) for 4 hours at 130°C. In a separate tube, 1 mL of chloramine T and 5 μL of skin hydrolysate were mixed and maintained at room temperature for 20 minutes. Then, 1 mL of perchloric aldehyde (15 g of dimethylaminobenzaldehyde, 60 mL of n-propanol, 26 mL of 60 % perchloric acid, and n-propanol to complete the volume to 100 mL) was added and stored at 60 °C for 15 minutes. Chloramine T is oxidized by hydroxyproline to pyrrole, which, in turn, reacts with perchloric aldehyde to form a red-purple complex, the absorbance of which could be measured at 550 nm. The quantification of samples was performed by interpolating the absorbance results obtained using the linear regression equation of the standard curve (y = 0.5972x + 0.0142/R = 0.99).

### Statistical Analysis

Data are expressed as mean ± standard error. The Student’s *t* test was employed to analyze data from the two groups. For analysis of the various groups and temporal procedures, we used ANOVA, followed by Bonferroni’s post-test. Linear regression was employed to design curves. *P*-values < 0.05 were considered statistically significant.

## RESULTS

### Analysis of Sesquiterpenes and Diterpenes Present in Copaiba Oil

The qualitative and quantitative analysis of oleoresin led to the identification of 24 peaks of sesquiterpenes and diterpenes that represented slightly more than 50 % of the total composition (Table 1). The major compounds found in the sample were the sesquiterpenes α-bergamotene (7.04 %) and β-caryophyllene (11.48 %). Other compounds frequently present in copaiba oleoresins in the range of 1 to 5% included copaene (1.61 %), α-curcumene (4.62 %), and β-humulene (3.05 %). The major diterpenes found in the copaiba oil were kaur-16-ene (1.4 %), kaurenoic acid (2.0 %), and cativic acid (2.25 %) (Table 1). 

**Table 1.** **Sesquiterpenes and Diterpenes identified in the Copaifera oleoresin, compared with the literature and fragmentation spectrum.** It was possible to identify peaks of 24 compounds that represent little more than 50 % of the total composition. The major compounds found were the sesquiterpenes α-bergamotene (7.04 %) and β-caryophyllene (11.48 %). Some compounds frequently presents in copaiba oleoresins were found at 1 to 5 %, including Copaene (1.61 %), α-curcumene (4.62 %) and β-humulene (3.05 %). The major diterpenes found in the copaiba oil were Kaur-16-ene (1.4 %), kaurenoic acid (2 %) and Cativic acid (2.25 %).

Compounds	Retention Index	Composition (%)
Cyclosativene	1125	0,82
Copaene	1221	1,61
α-cedrene	1403	4,18
α-zingiberene	1451	0,40
α-cubebene	1344	1,56
δ-selinene	1481	0,47
β-Caryophyllene	1494	11,48
α-bergamotene	1430	7,04
α-guaiene	1490	1,32
β-farnesene	1440	0,75
α-caryophyllene	1579	2,48
α-curcumene	1524	4,62
β-humulene	1574	3,05
γ-cadinene	1435	0,31
δ -cadinene	1469	1,39
Bergamotol	1673	0,58
α-bisabolene	1625	0,83
Guaiol	1614	0,27
Aromadendrene	1380	0,42
α-caryophyllene	1579	0,74
β-bisabolene	1619	0,3
Kaur-16-ene	1789	1,4
Cativic acid	2016	2,25
Kaurenoic acid	2050	2
Total of identified substances		50,27

### Minimum Necrotic Dose Determination (MDN)

The MDN was defined as 2.4 μg/kg, because this was the lowest venom dose capable of inducing a necrotic area of ​​at least 1 cm^2^ in 72 hours in 100 % of animals (data not shown).

### *L. intermedia* Venom Reduced Platelet and Heterophil Count

**Figure 1.**
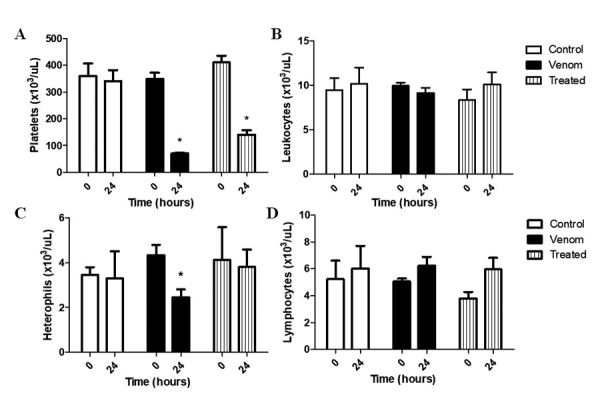
**Effect of treatment with copaiba oil after *Loxosceles intermedia* venom injection on blood cells.** Platelets **(a)** and heterophils **(c)** count showed significant reduction after venom injection (4,8 μg/kg). The treatment was efficient in inhibiting the decrease of heterophils in the blood 24 hours after venom inoculation *p <0.05 / ANOVA. However, groups inoculated with venom and treated with copaíba oil showed no significant difference from the control group at total leukocyte **(b)** and lymphocyte count **(d)**. n = 4 animals per experimental group.

A significant reduction in the number of platelets was observed in all groups inoculated with *L. intermedia* venom (4.8 μg/kg) as compared with the control group (Figure 1A). Although the number of total leukocytes did not differ significantly between the groups (Figure 1B), a notable reduction in the number of heterophils in the blood of the animals inoculated with *L. intermedia* venom was reported. Treatment of animals with copaiba oil curbed the reduction in heterophil count induced by spider venom (Figure 1C). No significant difference was observed between the groups analyzed with respect to lymphocytes (Figure 1D).

### Topical Treatment with Copaiba Oil and Effects on Cicatrization

**Figure 2.**
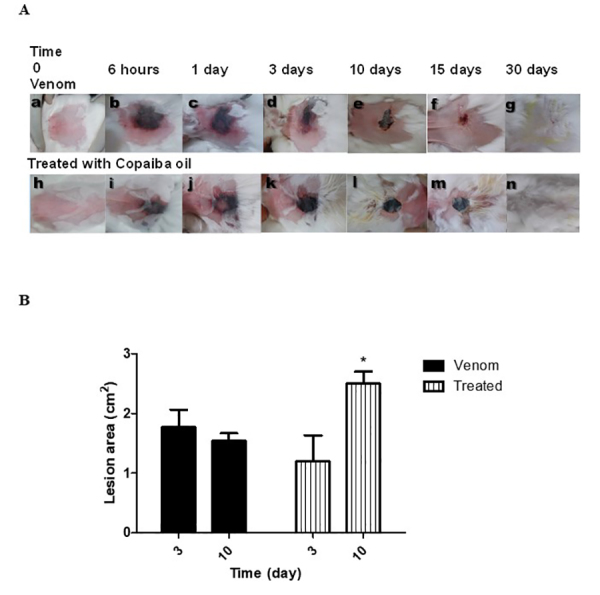
**Evolution of the lesion 30 days after *Loxosceles intermedia* venom injection.** Macroscopic comparison of animals that received only the venom, compared to those treated with copaiba oil after venom inoculation showed lesions with smaller area and with mild erythema. The copaiba oil induced a formation of thicker crust more delimited, without the presence of scarring on the skin **(a)**. Ten days after venom injection the lesion area of the group that received the treatment with copaiba oil was larger than the untreated group **(b)**. n = 4 animals per experimental group.

Rabbits were evaluated for the effects of venom injected intradermally (4.8 µg/kg). Macroscopic analysis of skin lesions formed after inoculation of venom revealed typical dermonecrosis in all animals, however with different healing profiles. Topical application of copaiba oil induced formation of a thick but limited crust at the site of venom inoculation, without the presence of scarring at 30 days after venom inoculation (Figure 2A). For the animals that received topical treatment with copaiba oil, smaller lesions with mild erythema were observed after 3 days. Ten days after venom injection, the lesion area of the group receiving copaiba oil treatment was larger than that of the untreated group (Figure 2B).

### *L. intermedia* Venom Induced Local Skin Inflammation, Muscular Necrosis, and Hemorrhaging

**Figure 3.**
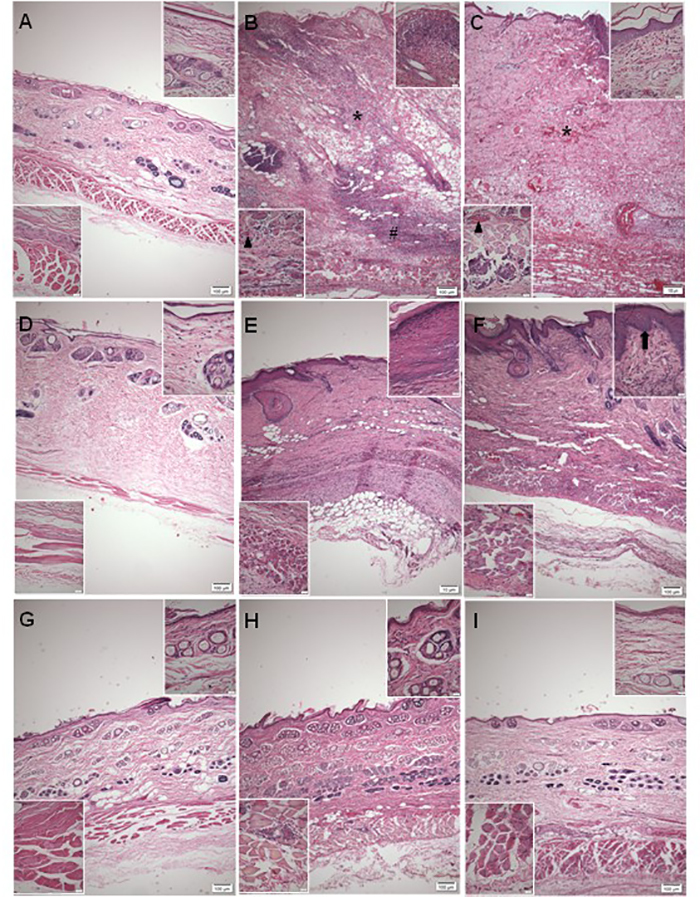
**Photomicrographs of the skin of rabbits 3, 10 and 30 days after *L. intermedia* venom inoculation in the back.** The control group presented integrity of the skin layers (**a/d/g**). In the animals that received venom injection (4.8 µg/kg) heavy bleeding in the dermis (*), epidermal thickening (upper insert - **b**), the presence of intense inflammatory infiltrate in muscle (lower insert - **b**) and hypodermis tissue (#) and blood vessels with clot ( ) were observed 3 days after inoculation **(b)**. In animals inoculated with venom and treated with copaiba oil, bleeding (*), injury of the epidermis (upper insert - **c**) and inflammatory infiltrates in muscle tissue (lower insert - **c**) were observed 3 days after venom inoculation (**c**). After 10 days, the dermal thickness was reduced in venom group (**e**), but the epidermis remained thick (upper insert - **e**); on the other hand, in the treated group, dermal thickness was reduced (**f**), but redefinition was detected between papillary and reticular dermis (upper insert - **f**). After 30 days of regeneration of the dermis, epidermis (upper insert - **h**) and muscle tissue, marked by the presence of central nuclei (inferior insert - **h**) after 30 days in the venom group (**h**); the treated group also showed regeneration of dermis, epidermis (upper insert - **i**) and muscle tissue (lower insert - **i**), marked by an increase in the number of hair follicles (**i**). n = 4 animals per experimental group.

For histopathological evaluation, the skins were removed from rabbits 3, 10, and 30 days after venom inoculation. In control animals, PSS had no effect on histopathological parameters on the mentioned days (Figure 3A, D, G). Animals inoculated with the venom showed heavy bleeding, thickening of dermis and epidermis, and presence of intense inflammatory infiltrate 30 days after injection (Figure 3B). These animals presented intravascular clots as well. The group inoculated with venom followed by treatment with copaiba oil also showed heavy bleeding, with inflammatory infiltrate and lesions on the epidermis (Figure 3C). On day 10 after venom injection, a significant inflammatory infiltrate in the muscular tissue was observed (Figure 3E, F). Furthermore, treated animals showed crucial tissue redefinition between papillary and reticular dermis (Figure 3F), an important histological aspect absent in the untreated group (Figure 3E). At day 30 after venom inoculation, dermis and epidermis had regained their integrity, with regeneration of muscle tissue and persistent inflammatory infiltrate (Figure 3H). Interestingly, animals inoculated with venom and treated with copaiba oil showed similarities with control groups, including integrity of all layers of the skin, high number of hair follicles, and sparse dermal inflammatory infiltrate (Figure 3G,I).

### Copaiba Oil Treatment Reduced Collagen Production

**Figure 4.**
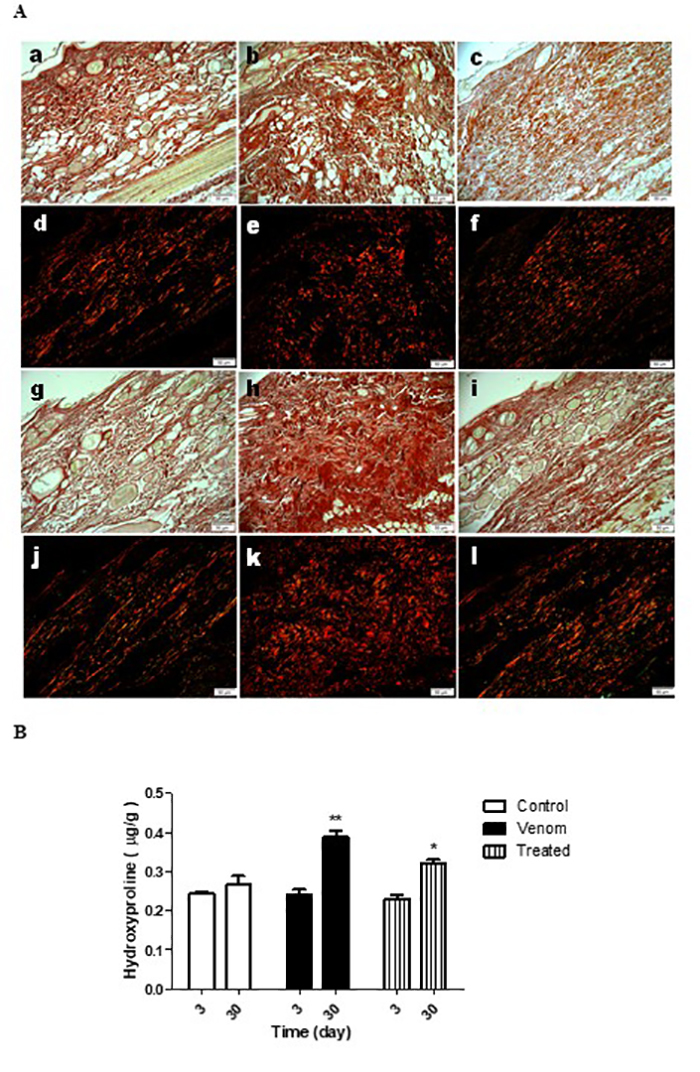
**Photomicrographs of collagen on skin of rabbits 3 and 30 days after inoculation of *L. intermedia* venom in the back.** Picrosirius red staining under optical light reveals total collagen as red, polarized light collagen type I as red fibers and collagen type III as green fibers. The control animals presented normal distribution of collagen types on the skin at 3 (**a** - a/d) and 30 days (**a** - g/j), and did not differ in total collagen quantification by hydroxyproline (**b**). The animals inoculated with venom at 4.8 µg/kg did not differ at day 3 post-inoculation compared to the control group (**a** - b/e) but showed increase of collagen deposition at 30 days (**a** - h/k), quantified by hydroxyproline (**b** *p<0.001 / ANOVA). The animal group inoculated with venom and treated with copaiba oil showed no difference at day 3 (**a** - c/f), but at 30 days presented less intense increase of collagen (**a** - i/l), quantified by hydroxyproline (**b** *p<0.05 / ANOVA). n = 4 animals per experimental group.

Optical microscopy analysis did not reveal significant differences with respect to collagen fiber deposition in all groups (Figure 4A, C). Polarized light microscopy was employed to analyze the distribution of collagen types I (red) and III (green) following picrosirius red staining. A large collagen deposit was detected in the venom-inoculated group 30 days after venom injection, in comparison with control and copaiba-treated animals (Figure 4G, I). Type I and type III collagen fibers were frequently observed in the control group (Figure 4J). The venom-induced significant changes in the dermis and type I collagen fibers were predominantly noted in this group (Figure 4K). Copaiba oil treatment restored the mixed profile of collagen fibers, such that both red and green stains were observed, similar to control groups (Figure 4L).

Total collagen was measured by quantification of hydroxyproline. All animals showed similar hydroxyproline levels on day 3 after venom exposure and respective treatments. However, 30 days after venom injection, hydroxyproline levels were significantly increased in comparison with the control group. Treatment with copaiba oil at this stage did reduce the hydroxyproline levels in comparison to the venom group although these were still elevated when compared with the control group (Figure 4M).

## Discussion

The oleoresin of *Copaifera* is widely used in Brazilian traditional medicine as an anti-inflammatory and healing agent [20]. However, evidence on its effectiveness and protective roles in treating injuries caused by *Loxosceles* spider bite is scarce. The results of the present study demonstrated the efficacy of topical application of copaiba oil in healing of dermonecrosis caused by injection of brown spider venom. We suggest that copaiba oil exerts its therapeutic effects on skin lesions induced by *L. intermedia* venom through various morphological changes, consequently leading to repair of damaged skin.

Distinct compounds identified in copaiba oleoresin are potentially responsible for some of the pharmacological properties, for example, β-caryophyllene, characterized by anti-inflammatory, antibacterial, anti-edema, antifungal, and antipsoriatic effects [21, 30, 31]. Other compounds present in copaiba oleoresin (viscous fluid) include kaurene and labdane diterpenoids, having broad-spectrum biological activities, as previously reported, including potential anti-inflammatory, antimicrobial, antitumor, and larvicidal properties [22, 28, 29].

Rabbit models show a high similarity to clinical events related to *Loxosceles* envenomation in humans, such as occurrence of intravascular coagulation causing occlusions of venules and arterioles that result in tissue hypoxia [32]. Intravascular coagulation can occur in the lungs, liver, and kidneys [7], when the venom reaches the systemic circulation. In severe cases, it may progress to death resulting from kidney failure [33]. The abrupt reduction in platelet count observed in venom-inoculated groups could not be countered by the application of copaiba oil after 24 hours. Reduced platelet count and increased fibrinogen synthesis are the first systemic responses of rabbits to injection of *Loxosceles* spider venom, approximately 12 hours after envenomation [12], a response very similar to that noted in humans.

Cellular changes in the bone marrow and peripheral blood of rabbits after exposure to *L. intermedia* venom have been reported. Thrombocytopenia is an important clinical sign during the diagnosis of *L. intermedia* spider envenomation [11]. Thrombocytopenia and a high number of heterophils were detected in the bloodstream of venom-inoculated rabbits, which were directly related to histopathological findings obtained from skin biopsies [34]. Evaluation of leukocyte mobilization and biochemical parameters in the blood of rabbits revealed that platelet functions and blood coagulation showed a time-dependent trend at 3, 24, 48, 72, and 120 hours after *Loxosceles* envenomation. These levels were associated with initial leukopenia and thrombocytopenia, posterior leukocytosis, platelet aggregation, elevation of fibrinogen levels, and reduction of coagulation factor VII [35]. Increase in leukocytes and heterophils occurred at 72 hours, the same time at which red blood cells declined [12].

On the other hand, in the current study, blood collected 24 hours after inoculation of spider venom from *L. intermedia* did not show significant differences in the cellularity of total leukocytes in any group. A significant reduction of heterophils was shown after venom injection but not after treatment with copaiba oil. Diminution in the number of circulating heterophils after 24 hours may be related to an acute inflammatory response, considering that these cells are found at the injury site within a few hours after venom injection and are reduced in blood circulation after 24 hours, as also observed in mice [1]. Together, these results indicated a lesser migration of heterophils to the skin and possible protective effect associated with reduced heterophilic response after venom injection and treatment with copaiba oil. These results imply that copaiba oil has the potential to partially control the inflammatory response, once heterophil levels in blood circulation decrease.

Histopathological analysis of the skin obtained from venom-inoculated rabbits demonstrated swelling of dermal endothelial cells [36], followed by deposition of intravascular fibrin, endothelial thickening, vasodilation and inflammatory cell infiltration, predominantly by polymorphonuclear cells [37]. Moreover, 24 hours after venom administration, a massive infiltration of leukocytes and platelets, bleeding, and thrombus formation at the site of venom inoculation were observed [35]. Long-term exposure to venom induces necrosis of myofibrils and infiltration of leukocytes, damaging the skeletal muscle. This, in turn, causes severe destruction of the epidermal integrity and necrosis of the connective tissue enriched with collagen fibers near the epidermis - the last events observed during the development of dermonecrotic injury in rabbits [5].

Wound healing is a complex process. Major shortcomings in this process may occur in the early stages, producing severe edema, reducing vascular proliferation, and decreasing cellular elements, such as leukocytes, macrophages, and fibroblasts [38]. Studies on the healing property of copaiba oil have concentrated on the resin phase owing to the presence of diterpenes, but cellular and molecular mechanisms of its potential therapeutic actions are poorly understood. In the present study, no acceleration of the healing process was observed in any animal group. However, the venom group demonstrated an adherent crust that broke away before 30 days of monitoring. This feature was not observed when animals received copaiba oil; in contrast, these animals showed a larger area of injury, at the 10^th^ day, which may plausibly account for the scar formation observed in the venom group.

The need for precision in tissue healing is impaired by the speed of repairing the tissue damage, without aggravating it. In this context, what occurs most often is the formation of scars, where the tissue will lose its function [39]. Thus, the ideal treatment of wounds should also value the quality of the healing process, for which the deposition of collagen is vital for recovering the lost cell mass in the lesion, thus refilling the damaged tissue. However, exaggerated deposition of collagen can cause fibrosis and impair the formation of functional tissue [40]. After 30 days, the animals treated with copaiba oil presented a distribution of collagen fibers more similar to the control group, which demonstrates a higher quality of healing. If the distribution of collagen is closer to that in the control, it is indicative that the skin is more resistant to possible lesions, thus exerting its function of protecting the organism. Our results demonstrated that copaiba oil promoted the regeneration of type III collagen fibers, with a collagen-fiber distribution similar to the normal skin of the animal. It can be inferred that copaiba oil may improve the process of skin regeneration, but further studies should be done to evaluate the structure of this tissue.

## Conclusions

The increasing number of accidents caused by brown spider bites in recent years has become a major public health concern in Brazil. Thus, the study of the actions of both venom and drugs has gained extreme importance to better manage envenomation and to provide proper treatment. The present work demonstrated the potential of copaiba oil to interfere in and curb the progression of spider-bite-induced dermonecrosis. The results described herein suggest that treatment with copaiba oil may modify scarring via deposition of collagen, thereby stimulating the growth of hair follicles and regeneration of muscle tissues. In addition, we now report that copaiba oil efficiently inhibits the heterophil reduction in the blood after venom injection. In light of the broad-spectrum applications of copaiba oil and its economic importance in Brazil, the present study encourages further research to elucidate its effects on wound healing.

## Abbreviations

 Not applicable.
